# Beneficial Effects of Traditional Chinese Medicine in the Treatment of Premature Ovarian Failure

**DOI:** 10.1155/2022/5413504

**Published:** 2022-11-26

**Authors:** Ming Li, Yu-Bo Xiao, Le Wei, Qi Liu, Pin-Yue Liu, Jian-Feng Yao

**Affiliations:** ^1^Department of Histology and Embryology, Hunan University of Medicine, Huaihua, China; ^2^Quanzhou Maternity and Child Healthcare Hospital, Quanzhou, China

## Abstract

Premature ovarian failure (POF) is characterized by hormonal disorders, amenorrhea, and premature loss of fertility potential in women of reproductive age. Several studies have been conducted on the effectiveness of traditional Chinese medicine (TCM) in treating POF. TCM relied primarily on apoptosis, immunity, and aging to treat POF based on the studies of domestic and foreign literature. Zuogui pills inhibited mitochondrial-dependent apoptosis in the treatment of POF. Huyang Yangkun formula regulated the downstream of the Bcl-2 family to resist apoptosis through the aquaporin-1 protein. Modified Bazhen decoction regulated apoptosis in POF by regulating X-linked inhibitors of apoptosis protein. Bushen Tianjing recipe was effective in treating POF by promoting angiogenesis and preventing apoptosis. As for immunity, Bushen Jianpi prescription and Er-Xian decoction cured autoimmunity POF models and increased follicular development-related protein expression. Bushen Huoxue Tang improved ovarian function and reduced ovarian inflammation by regulating the Nrf2/Keap1 signaling pathway and T lymphocytes. Taohong Siwu decoction promoted the proliferation and differentiation of granulosa cells of POF mice by regulating the TGF-*β*1/Smads signaling pathway. In addition, ginsenoside Rg1 and Jiajian Guisheng formula treated POF by regulating cell aging-related mechanisms. Si Wu Tang treated POF by activating the angiogenesis-related proteins. The goal of this review is to serve as a reference for in-depth research into the treatment of POF with TCM and provide inspiration for new diagnostic methods and treatment options.

## 1. The Current State of POF Research 

Premature ovarian failure (POF) or premature ovarian insufficiency (POI) can diminish female fertility potential prematurely. Symptoms of POF include cessation of menstruation, follicular dysplasia, and disruption of elevated follicle-stimulating hormone (FSH), estradiol (E2), anti-Müllerian hormone (AMH), and luteinizing hormone (LH) levels in women before forty [1]. Likewise, it is also accompanied by hot flashes, night sweats, insomnia, psychological distress, sexual dysfunction, and other symptoms similar to menopause. Chemotherapy, autoimmunity, and genetic defects could contribute to POF's etiology [2–4]. Mauri et al. conjectured that chemotherapy might damage DNA, inducing early cell apoptosis or premature follicles [5]. Chromosomal abnormalities are considered a common cause of POF. According to Jin et al., the coding variants ADAMTSL1, FER1L6, and the minichromosomal maintenance complex components (MCM) 8 and MCM 9 were also linked with POF [6].

POF is difficult to prevent and cure. Estrogen therapy and progesterone therapy are used for clinical treatment. Estrogen has been reported to avoid the adverse effects of clomiphene citrate and apoptosis in the ovarian follicles of the mammalian ovary [7]. Melatonin is helpful in the treatment of chemotherapy-induced ovarian dysfunction by reducing oxidative stress levels and preventing apoptosis in the ovarian follicles and oocytes [8–11]. However, hormone therapy may cause side effects such as obesity, headaches, and even malignant tumors. Therefore, it is necessary to look for alternative forms of treatment. The transplantation of stem cells could stimulate the growth of follicles and improve hormone levels. In the study of Fu et al., overexpression of miRNA-21 in mesenchymal stem cells led to the inhibition of granulosa cell (GC) apoptosis and improved ovarian structure and function in chemotherapy-induced POF rats [12]. Ding et al. determined that human umbilical cord mesenchymal stem cell-derived exosomal miRNA-17-5p enhanced ovarian function in POF mice by regulating Sirtuins7 [13]. Even so, stem cells still have limited clinical applications. Besides the high cultivation costs and limited supply of stem cells, stem cells are also subject to immune rejection and ethical concerns in patients.

## 2. Application of Traditional Chinese Medicine in POF

Traditional Chinese Medicine (TCM) has long been used in China for the treatment of a wide variety of illnesses. Herbal medicine resources in China are abundant, easy to grow, and affordable. Different therapeutic effects could be achieved by mastering different drug combinations. TCM could improve the body's resistance. Studies suggested that TCM possessed the therapeutic potential for the treatment of neurological disorders, cancer, and COVID-19 [14–16]. Menstruation and ovulation could be improved with TCM, as well as perimenopausal symptoms such as hot flashes, sweating, insomnia, anxiety, and osteoporosis [17]. TCM is effective in the treatment of several gynecological disorders such as polycystic ovary syndrome and infertility [18, 19].

POF was described based on its symptoms as “amenorrhea,” “infertility,” or “menstrual water loss at a young age” among ancient Chinese medical texts. Kidney deficiency (Shen Xu) was one of the primary causes of POF in TCM [20]. Qu et al. asserted that kidney deficiency was the origin, followed by liver stagnation and spleen deficiency based on the etiology and pathogenesis of POF [21]. In addition to strengthening the kidneys and feeding the essence, it was also necessary to maintain the spleen and soothe the liver to treat POF. Several studies and rich clinical experiences had demonstrated that TCM prescriptions were beneficial in treating POF. Zigui Yijing decoction combined with Zishen Yutai pills reduced the level of FSH and LH and increased the level of E2 and AMH. They improved the ovarian blood supply and the thickness of the endometrium in POF patients. With combined medication, POF patients' symptoms such as insomnia, hot flashes, night sweats, and dry mouth and throat were improved [22]. The total effective rate of the treatment groups was 63.6%. In a network pharmacology study, Zigui Yijing decoction activated PI3K/Akt signal pathways by affecting IL-6, AKT1, and PTEN, thus treating POF [23]. Xie et al. reported that the Huyang Yangkun formula repaired dysfunctions in POI rats through the Hippo-JAK2/STAT3 pathway [24]. Keremu et al. reported that TCM Muniziqi improved the function of the hypothalamus-pituitary-ovarian axis in POF rats [25]. TCM could treat POF that is caused by apoptosis or immunosuppression [26, 27].

### 2.1. TCM Prevent POF by Inhibiting Apoptosis

Primal follicles (PF) or ovarian reserve are fixed at birth and activated by the PI3K/AKT/mTOR pathway and the Hippo signaling pathway [28]. The majority of follicles eventually develop into the corpus luteum. However, 99% of follicles develop follicular atresia, which is vital for the maintenance of a healthy and functioning reproductive system. GC plays a vital role in deciding follicular fate, providing molecules essential for follicle growth, as well as dying off through apoptosis to result in follicular atresia [29]. The division, proliferation, and apoptosis of GC coexist under normal circumstances, but follicular atresia occurs when GC apoptosis >10% [30]. At the same time, once the process of follicular atresia becomes out of control, it would accelerate apoptosis, which causes premature PF depletion [31]. Autophagy induces apoptosis by triggering the accumulation of autophagosomes in the GC and luteal cells, also leading to follicular atresia [32]. The rate of follicle atresia is greater than normal physiological metabolism, which can lead to POF. Apoptosis is caused by oxidative stress (OS). Pathological conditions and drug treatments generate OS in the ovary and induce GC apoptosis through mitochondria-cytochrome C (Cyt-c) pathway [33]. Cyt-c binds to caspase factor 1, activating caspase-9 in the oocyte cytoplasm [34]. Active caspase-9 triggers the conversion of procaspase-3 to caspase-3 [35]. Furthermore, activation of caspase-3 disrupts cellular organization and ultimately leads to oocyte apoptosis [36]. TCM had been demonstrated to affect apoptosis factors in POF in recent years (Table 1).

Zuogui Pill (ZGP) is a classic TCM prescription used to treat POF. In ZGP, Shan Yao has the effect of nourishing the spleen “yin.” Gou Qi Zi has the effect of tonifying the kidney and nourishing essence, clearing the liver. Shan Zhu Yu could nourish the liver and kidneys. Gui Jia Jiao could replenish the “yin” of the liver and kidney. Lu Jiao warms the kidney “yang,” astringents, and stops bleeding. Bian et al. found that clinical treatment of POF with ZGP could reduce the occurrence of adverse reactions (dizziness, thrombosis, etc.), improve the hormone level of patients, and promote body recovery [40]. ZGP inhibited GC apoptosis in rats and PI3K/AKT/mTOR pathway activation improved ovarian function induced by cisplatin [41]. ZGP induced the expression of the TAP63 protein in ovarian tissue, activated the Bcl-2 gene, and inhibited GC and oocyte apoptosis [42]. Serum FSH concentrations decreased and serum E2 levels increased with ZGP treatment. Compared with the model group, the pregnancy rate was improved but not statistically significant, the number of primary follicles, second follicles, and corpus luteum increased significantly, and the number of atresia follicles decreased after the administration of ZGP. Moreover, Bax and Cyt-c were repressed, and Bcl-2 increased. Their results suggested that ZGP-regulated inhibitory mitochondrial apoptosis in follicles had a significant impact on POF [26].

Huyang Yangkun formula (HYF) is based on the “yin and yang” theory, which suggests that POF may be caused by an imbalance of the kidney “yin and yang”. HYF regulated JNK/p38/p65-NF*κ*B pathway, reduced the apoptosis of GC, and protected the ovary [43]. In Lingdi Wang's experiment, with HYF treatment, AMH and Bcl-XL were increased, aquaporin 8 (AQP8), cleaved-caspase-9, and cleaved-caspase-3 were downregulated, and the number of apoptotic cells significantly decreased. The follicles developed variously, the microvessels were abundant, and the number of follicles was significantly increased in the HYF group. Their results indicated that HYF promoted follicle development by modulating mitochondrial apoptosis [37].

Modified Bazhen decoction (MBD) was used for treating postpartum blood deficiency, viscera deficiency, and excessive sweating. Yang et al. found that MBD could improve POF by inhibiting excessive autophagy of ovarian cells [44]. In Liu et al.'s study, E2 and X-linked inhibitors of apoptosis protein (XIAP) were increased, and FSH was decreased with MBD treatment [38]. XIAP could bind to the apoptosis initiator caspase-9, and apoptosis effectors caspase-3 and caspases-7, to down-regulate apoptosis [45]. Therefore, Liu speculated that MBD could regulate apoptosis and play a therapeutic role in POF by regulating XIAP.

Bushen Tianjing recipe (BTR) had been used for centuries in treating POF. The menstruation recovery rate of POF patients was 91.18% after half a year of treatment with BTR. There was no liver and kidney function damage or other complications, which proved that BTR had good clinical efficacy [46]. Xu et al.'s study showed that BTR decreased the estrous cycle, and increased the ovary index. BTR treatment significantly reduced the histopathological changes of POF and increased the number of primordial follicles and primary follicles. It reduced ovarian interstitial fibrosis, degenerative necrosis of the corpus luteum, inflammatory cell infiltration, and vasodilation symptoms. Also, BTR elevated E2 and decreased FSH, progesterone, and testosterone. BTR increased the level of Bcl-2, decreased the level of Bax and caspase-3, and protected GC from apoptosis [39].

DNA damage is the hallmark feature of apoptosis. The DNA damage and PI3K/AKT pathway are involved in follicle survival. Lacking CK2*β* in oocytes enhanced *γ*H2AX, a member of the histone H2A family, suppressed PI3K/AKT signaling, and impaired DNA damage response signaling, leading to POF [47]. Icariin is an extract of Yin Yang Huo. Li et al. demonstrated that icariin promoted DNA repair and protected GC and follicles in POF [48]. The levels of *γ*H2AX and p53 binding protein 1, indicators of DNA damage, decreased in POF mice after icariin treatment. Icariin increased body weight and ovarian mass, increased the content of serum E2, and decreased the content of FSH in POF mice. Icariin increased the number of primordial follicles, primary follicles, and mature follicles in POF mice. Icariin downregulated the expressions of MafG and Gcnt3 genes and upregulated the expressions of Oas1h, Smc1b, Mov10l1, Oosp2, Tbpl2, and DynAP genes in POF mice, participated in DNA repair [49]. Their results demonstrated that icariin attenuated cell apoptosis by promoting DNA damage repair.

### 2.2. TCM Improves POF through Immunity

The immune system fails to identify the ovary's cellular self correctly in 10%–30% of POF patients. They suffer from autoimmune diseases, such as hypothyroidism, systemic lupus erythematosus, rheumatoid arthritis, and Crohn's disease [50]. An auto-inflammatory process could damage the ovary by altering T cell subsets, increasing antioocyte antibodies and antiovarian antibodies produced by B cells. The Auto-inflammatory process could decrease the number of effector-suppressed/cytotoxic lymphocytes and promote natural killer (NK) cell activity [51]. Lipid oxidative stress injury and polarization of immune cells are inhibited and the release of inflammatory factors is increased in POF mice. Zhu et al. reported that the concentrations of IL-1*β*, IL-6, IL-4, IL-22, NF-*κ*B, TNF-*α* and IFN-*γ* in the peripheral blood of POF mice were significantly decreased after thymopentin treatment [52]. Yin et al. reported that Th17/Tc17 cells, Th17/Treg cells, or IL-17 expression were increased, restoring ovarian function after human placenta mesenchymal stem cells were transplanted into POF mice [53]. TCM had been demonstrated to affect the immune in POF in recent years (Table 2).

POF patients suffering from autoimmune are not appropriate candidates for gonadotropin therapies, which would accelerate follicular turnover, reduce the effectual follicle reserve in PF and aggravate the disease [51]. Therefore, many researchers focused on studying TCM curing POF. He et al. treated POF mice with Ginsenoside Rg1 (Rg1) extracted from ginseng. Rg1 treatment significantly reduced the expression of malondialdehyde, IL-1*β*, TNF-*α,* and IL-6 [58]. Rg1 improved fertility and reduced ovarian pathology in POF mice by enhancing anti-inflammatory and antioxidant functions.

Bushen Jianpi prescription (BJP) is derived from the formula used to prepare Shuangbu decoction, which was originally documented in medical literature “Wenbing Tiaobian” authored by Tang Wu in the Ming dynasty [59]. Feng et al. used BJP to treat autoimmune POF mice. Compared with model groups, ovarian weights in BJP treatment groups were significantly increased. The levels of E2, LH, and FSH were improved and the expressions of the antibone morphogenetic protein 15 (BMP-15) and connexin 43 (Cx43) were increased after BJP treatment [27]. Cx43 regulates folliculogenesis and oogenesis, and BMP-15 positively regulates follicle growth and differentiation, oocyte development, ovulation, fertilization, and embryonic development [60]. Therefore, BJP had a therapeutic effect on autoimmune POF mice and improved folliculogenesis, oogenesis, and follicular development. Tian found that the hormone level in the POF patient's body was significantly improved with BJP. The levels of CD4^+^ T cells and CD4^+^/CD8^+^ of T cells in POF patients increased significantly, while the levels of CD8^+^ T cells decreased significantly after BJP treatment [61]. Therefore, BJP could cure POF by regulating immunity.

The Er-Xian decoction (EXD) is mainly recommended to treat menopause, amenorrhea, and other symptoms related to “Shen Xu”. In EXD, Ba Ji Tian can invigorate the kidney and strengthen the “yang.” Zhi Mu and Huang Bai can purge the kidney “yang” and nourish the kidney “yin”. According to the system pharmacology analysis, EXD might exert its beneficial effects by modulating immune activity, estrogen levels, and antioxidant activity on POF [54]. The study of traditional Chinese medicine gynecology in Xi'an, China showed that the clinically effective rate of EXD was 91.3%. In the EXD treatment group, the indexes such as mean ovarian diameter, ovarian average volume, and oral follicle number were significantly increased, and the endocrine hormones were significantly improved. The levels of CD4^+^ T cells and CD4^+^/CD8^+^ of T cells in POF patients increased significantly, while the levels of CD8^+^ T cells decreased significantly after EXD treatment [62]. In the experiment of Zhang et al., EXD effectively treated autoimmune POF mice. EXD significantly improved the average weight of the ovary, spleen, and thymus of autoimmune POF mice, but had no significant difference in the overall weight. EXD treatment significantly increased CD3^+^ T cells and CD4^+^ T cells and decreased CD8^+^ T cells. The levels of serum LH and FSH declined and the level of E2 increased, the expression of BMP-15 and Akt were higher after being treated with EXD [63].

In Bushen Huoxue formula (BHF), Dang Gui and Chuan Xiong have the function of nourishing blood and regulating menstruation. Di Huang and Bai Shao can nourish “yin,” blood, and liver. Tu Si Zi can help “yang” and replenish essence. Yin Yang Huo can invigorate the kidney and strengthen the “yang.” BHF had been used clinically to treat POF. Wang et al. from Qinhuangdao Maternal and Child Health Hospital found that BHF could effectively reduce the abnormal ovarian immune response in POF patients, and the levels of CD3^+^ T cells, CD4^+^ T cells, and CD4^+^/CD8^+^ T cells were reduced in peripheral blood. BHF improved the estrogen level in POF patients. The effective rate of BHF treatment was 82.42%, higher than 60.44% in the control hormone treatment group [64]. Chen et al. demonstrated that BHF improved body weight, ovarian and spleen indexes index, ovarian function, and estrous cycle by Nrf2/Keap1 signaling pathway in the autoimmune POI model. BHF significantly increased E2 and AMH levels and decreased FSH and LH levels. It also increased SOD levels and decreased malondialdehyde levels, ameliorating oxidative stress in POF mice [55]. Wang et al. found that BHF exerted significant protective immunity on POF mice by regulating T lymphocytes [56]. BHF stimulated the expansion of CD4^+^ T cells, CD25^+^ T cells, and Fox P3^+^ (forkhead box P3^+^) T cells in autoimmune POF mice, the levels of IL-10 and IFN-*γ* were decreased. The body weight and multiple organs index of the BHF-treated group increased. The number of developing follicles was greater. The expression of estrogen and progesterone receptors (ER and PR) also increased with BHF treatment.

Yuan et al. found that modified Taohong Siwu decoction (TSD) increased the number of primary and mature follicles and decreased the number of atretic follicles in immune POF model mice [57]. Meanwhile, TSD improved ovarian function by up-regulating the expression of TGF-*β*1, TGF-*β* RII, and Smad 2/3 proteins in GC. Thus, TSD promoted the proliferation and differentiation of GC of POF mice by the TGF-*β*1/Smads signaling pathway. TGF-*β* is a master regulator of multiple cellular functions, including cellular immunity, as well as an important executor of immune homeostasis and tolerance [65]. Besides regulating the production and function of effector T cells and dendritic cells, TGF-*β* could suppress NK cells and regulate the complex behavior of macrophages and neutrophils [66].

### 2.3. TCM Treats POF through Other Ways

Besides apoptosis and immunity, there are several other pathogenic factors of POF, including the complex microenvironment of the ovary and cell aging (Table 3). The ovarian microenvironment contains estrogen and growth factors related to follicular development, such as Nrf2, Akt, NF-*κ*B, and multiple interleukins [69, 70]. Follicular development and ovulation are related to ovarian blood vessels [71]. VEGF polymorphisms are significantly associated with POF in patients [72]. Hence, remodeling the vascular system is essential for ovarian function. Known as “the first prescription for gynecology,” Si Wu Tang (SWT) was widely used as an effective remedy for menstrual irregularities and infertility. In the research of Zhou et al., the highest-dose SWT group significantly increased the pregnancy rate and improved the number of live births and the weight of newborn mice. They found that SWT treatment significantly improved estrogen levels, follicle numbers, antioxidant defense, and microvascular formation in POF mice by promoting activation of the STAT3/HIF-1*α*/VEGF signaling pathway and Nrf2/HO-1 signaling pathway [67].

Reducing the premature aging of the follicles is also crucial to treating POF. P16^INK4a^ is a typical senescence marker and has been used exclusively in cancer diagnosis [73]. Long-term expression of p16^INK4a^ inhibits the growth of underlying cancer cells but also causes irreversible cell cycle arrest [74]. Sirtuin-1 (SIRT1), an NAD^+^-dependent enzyme, is deeply involved in apoptosis, autophagy, senescence, and proliferation [75]. SIRT1 is implicated in aging through various aging-related signaling networks, including NF-*κ*B, mTOR, P53, PGC1*α*, and FoxOs [76]. Liu et al. found that the TCM ginsenoside Rg1 increased the number of follicles, decreased the follicles in the corpus luteum, and improved hormone levels. Their research also demonstrated that Rg1 delayed D-gal-induced ovarian failure in POF mice by downregulating the expression of p16^INK4a^ and enhancing the expression of SIRT1 [77].

Senescence-associated heterochromatic foci (SAHF) is a unique phenomenon of senescent cells and the sign of aging. SAHF stands for punctate aggregated heterochromatin in the nucleus of senescent human diploid fibroblasts [78]. Genes encoded by this domain are repressed, promoting cellular senescence [79, 80]. Su et al., [68] treated POF mice with Jiajian Guishen formula (JGF) and significantly improved the estrous cycle [68]. E2 and Inhibin B (INHB) levels were elevated in serum with JGF treatment. JGF also inhibited the expression of SAHF-related proteins in high mobility group A (HMGA1, HMGA2) and reduced the distribution of HP1 and H3K9me3 in the ovary. Therefore, they speculated that JGF might save POF mice by inhibiting the SAHF process. SAHF, p16^INK4a^, and SIRT1 coregulated senescence and cell cycle arrest-related proteins in neuronal senescence [81]. There may also be an interaction between the three that contributes to the premature aging of cells in POF.

## 3. Future Perspective and Conclusion

**Figure 1:**
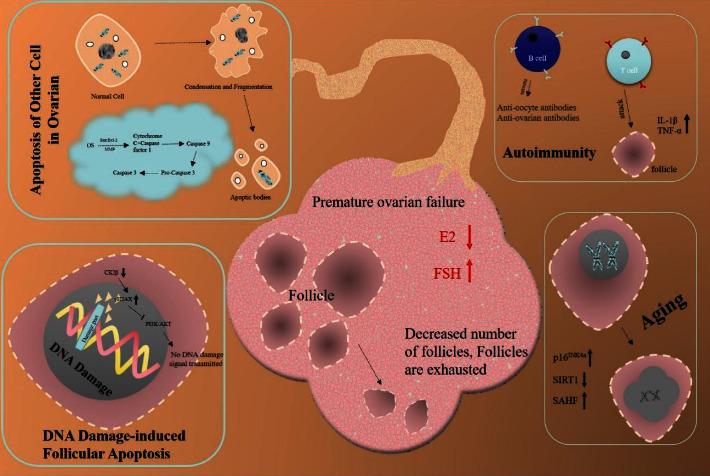
Multiple main factors contribute to POF.

**Figure 2:**
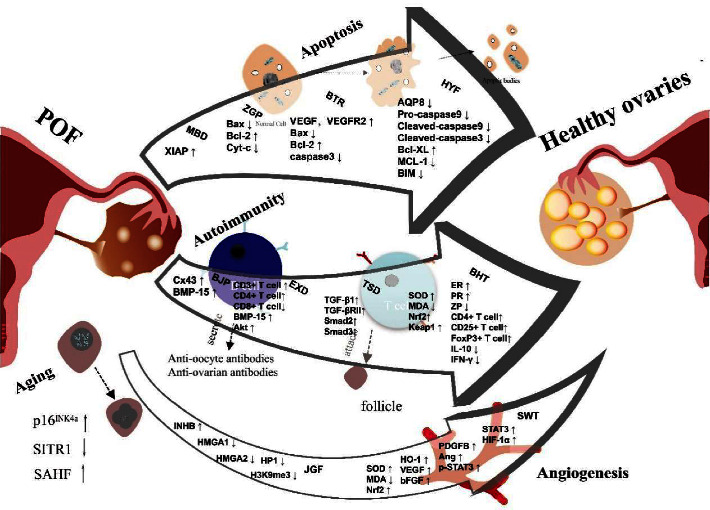
Mechanism diagram of multiple TCM prescriptions curing POF.

POF is caused by the interaction between genetic mutations caused by complex environmental factors and fast-paced lifestyles. POF has many pathogenic factors, including heredity, premature follicle aging, apoptosis, as well as effects of the patient's immune system on the ovary (Figure 1). Many TCM prescriptions had been proven effective in treating POF in China. According to TCM, POF is originating from deficiencies of the kidneys or abnormal liver and spleen functions. TCM treatments aim to invigorate the kidneys, soothe the liver, and strengthen the spleen. A variety of treatment methods are available, such as individual treatment based on differentiation of syndrome, special prescriptions plus or minus symptoms, and the cycle therapies of “yin and yang”. In this review, we summarized the related mechanism of TCM in the treatment of POF. Studies had shown that TCM prescription had therapeutic benefits by inhibiting apoptosis, slowing the aging process, promoting angiogenesis, and improving immunity (Figure 2). In the clinical and animal research, we cited in this paper, there was no mention of the phenomenon that the experimental individual had adverse reactions due to the toxicity of the prescription.

The most appropriate treatment for POF in TCM is still a matter of discussion and debate in clinical practice, as there are many methods of treating this disease. Apart from TCM prescriptions, there are also TCM prescriptions combined with acupuncture, enema, foot baths, and other forms of treatment. In addition to the TCM prescriptions in this review, others also had been proven to be effective for POF patients. But the total number of POF with TCM cases is small and scattered, and every patient's situation is different, clinicians fail to collect the necessary data in the usual treatment process for comparison. Therefore, the relevant clinical experimental articles of various prescriptions mentioned in this review were limited. Thus, we call on doctors to actively collect data and publish more excellent papers to prove the role of TCM. We also urge doctors to promote the application of TCM when treating POF patients.

## Figures and Tables

**Table 1:** The regulatory effects of Chinese herbal formula on POF through apoptosis.

Formula name	Formula	Hormone and related protein levels	Experimental model	Roles
Zuogui pills [26]	*Rehmanniae radix praeparata* (Shu di Huang), *Corni fructus* (Shan Zhu Yu), *Dioscoreae rhizoma* (Shan Yao), *Cuscuta chinensis* (Tu Si Zi), *Lycium barbarum* (Gou Qi Zi), *Achyranthes bidentata* (Niu Xi), *Testudinis carapax et plastic collar* (Gui Jia Jiao), *Cervi conrnus colla* (Lu Jiao Jiao) with a 8 : 4 : 4 : 4 : 4 : 3 : 4 : 4 ratio	FSH ↓	SPF female SD rats	ZGP inhibited mitochondrial-dependent apoptosis in the treatment of POF.
		E2 ↑		
		Bax ↓		
		Bcl-2 ↑		
		Cyt-c ↓		

Huyang Yangkun formula [37]	*Astragali radix* (Huang Qi) 50 g, *Herba epimedii* (yin yang Huo) 10 g, *Dioscoreae rhizoma* (Huai Shan Yao) 10 g, *Semen cuscutae* (Tu Si Zi) 10 g, *Rehmanniae radix* (Shu Di Huang) 10 g, *Angelicae sinensis radix* (Dang Gui) 10 g, *Glehniae radix* (Bei Sha Shen) 10 g	AMH ↑	SPF female SD rats	HYF regulated the downstream Bcl-2 family to resist apoptosis through AQP protein.
		FSH ↓		
		AQP8 ↓		
		Procaspase-9 ↓		
		Cleaved-caspase-9 ↓		
		Cleaved-caspase-3 ↓		
		Bcl-XL ↑		
		MCL-1 ↓		
		BIM ↓		

Modified Bazhen decoction [38]	*Bupleurum* (Chai Hu) 6 g, Angelica (10 g), *Ligusticum Chuan Xiong* (Chang Xiong) 6 g, Root of *Herbaceous penoy* 10 g, *Rehmannia* (Di Huang) 10 g, *Codonopsis* (Dang Shen) 10 g, Ctractylodes 6 g, Poria (Fu Ling) 10 g, Licorice (Gan Cao) 3 g, Antlers (Lu Rong) 10 g, Cyathula (Bei Xian) 20 g, Cyperus (Suo Cao) 10 g	FSH ↓	SPF female SD rats	MBD could regulate apoptosis in POF by regulating XIAP.
		E2 ↑		
		XIAP ↑		

Bushen Tianjing recipe [39]	*Rehmanniae radix praeparata* (Shu Di Huang) 10 g, *Testudinis carapax et plastrum* (Gui Jia) 10 g, *Paeoniae radix alba* (Bai Shao) 10 g, *Corni fructus* (Shan Yu Rou) 10 g	FSH ↓	SPF female SD rats	BTR was effective in the treatment of POF rats by angiogenesis and antiapoptosis.
		E2 ↑		
		Progesterone ↓		
		Testosterone ↓		
		VEGF, VEGFR2 ↑		
		Bax ↓		
		Bcl-2 ↑		
		Caspase-3 ↓		

**Table 2:** The regulatory effects of Chinese herbal formula on POF through the immune system.

Name	Formula	Hormone and related protein levels	Experimental model	Roles
Bushen Jianpi prescription [27]	*Radix rehmanniae* (Di Huang) 10 g, *Semen nelumbinis* (Lian Zi) 10 g, *Rhizoma dioscoreae oppositae* (Shan Yao) 10 g, *Semen cuscutae* (Tu Si Zi) 10 g, *Radix morindae officinalis* (Ba Ji Tian) 10 g, *Fructus rubi chingii* (Fu Pen Zi) 10 g, *Rhizoma polygonati sibirici* (Huang Jing) 10 g, *Herba dendrobii nobilis* (Shi Hu) 10 g, *Folium citri reticulatae* (Ju ye) 10 g, *Rhizoma cyperi* (Xiang Fu) 10 g and *Radix glycyrrhizae* (Gan Cao) 3 g	FSH ↓	Autoimmune POF model female BALB mice	BJP cured the autoimmune POF model and increased the expression of follicular development-related proteins.
		E2 ↑		
		LH ↓		
		Cx43 ↑		
		BMP-15 ↑		

Er-Xian decoction [54]	*Rhizoma curcumae phaeo-caulis* (E Zhu) 9 g, *Herba epimedii brevicornus* (yin yang Huo) 9 g, *Radix morindae officinalis* (Ba Ji Tian) 9 g, *Radix angelicae sinensis* (Dang Gui) 9 g, *Cortex phellodendri amurensis* (Huang Bai) 6 g and *Rhizoma anemarrhenae* (Zhi Mu) 6 g	FSH ↓	Autoimmune POF model female BALB/C mice	EXD relieved ovarian function of autoimmune POF model mice.
		E2 ↑		
		LH ↓		
		CD3+ T cell ↑		
		CD4+ T cell ↑		
		CD8+ T cell↓		
		BMP-15 ↑		
		Akt ↑		

Bushen Huoxue Tang [55, 56]	*Rehmannia glutinosa libosch* (Di Huang) 10 g, *Epimedium brevicornu Maxim* (yin yang Huo) 10 g, *Cuscuta chinensis Lam* (Tu Si Zi) 10 g, *Uncaria rhynchophylla* (Gou Teng) 6 g, *Angelica sinensis* (Dang Gui) 10 g, *Paeonia lactiflora Pall* (Bai Shao) 10 g, *Ligusticum chuanxiong Hort* (Chuan Xiong) 10 g, *Anemarrhena asphodeloides* Bge (Zhi Mu) 10 g, *Bupleurum chinensis* DC (Chai Hu) 6 g, *Phellodendron chinensis schneid* (Huang Bai) 10 g, *Scutellaria baicalensis georgi* (Huang Qin) 6 g	FSH ↓	Autoimmune POF model SPF female C57BL/6J mice	BHT improved ovarian function and reduced ovarian Inflammation by Nrf2/Keap1 signaling pathway.
		E2 ↑		
		LH ↓		
		AMH ↑		
		SOD ↑		
		MDA ↓		
		Nrf2↑ Keap1 ↑		
	*Rehmannia glutinosa libosch* (Di Huang) 10 g, *Epimedium brevicornu Maxim* (yin yang Huo) 10 g, *Cuscuta chinensis Lam* (Tu Si Zi) 10 g, *Angelica sinensis* (Dang Gui) 10 g, *Paeonia lactiflora Pall* (Bai Shao) 10 g, *Ligusticum chuanxiong Hort* (Chuan Xiong) 10 g, *Anemarrhena asphodeloides* Bge (Zhi Mu) 10 g, *Bupleurum chinensis* DC (Chai Hu) 6 g, *Phellodendron chinensis Schneid* (Huang Bai) 10 g	ER ↑	Autoimmune POF model B6AF1 female mice	BHT exerted significant protective immunity on POF mice by regulating T lymphocytes.
		PR ↑		
		ZP ↓		
		CD4+↑		
		CD25+↑		
		FoxP3+ ↑		
		IL-10 ↓		
		IFN-*γ* ↓		

Taohong Siwu decoction [57]	*Rehmanniae radix praeparata* (Di Huang) 15 g, *Polygonatum sibiricum* Red. (Huang Jing) 12 g, *Cornus officinalis Sieb. et Zucc*. (Shan Zhu Yu) 12 g, *Lycium barbarum* L. (Gou Qi Zi) 12 g, *Angelica sinensis* (Oliv.) Diels (Dang Gui) 15 g, *Paeonia lactiflora* Pall. (Bai Shao) 12 g, *Ligusticum chuanxiong* Hort. (Chuan Xiong) 9 g, *Salvia miltiorrhiza* Bge. (Dan Shen) 12 g, and *Prunus persica* (L.). Batsch (Tao Ren) 9 g	TGF-*β*1↑	Immune POF model female BALB/c mice	TSD promoted the proliferation and differentiation of granulosa cells of POF mice by the TGF-*β*1/Smads signaling pathway.
		TGF-*β*RII↑		
		Smad2↑		
		Smad3↑		

**Table 3:** The regulatory effects of Chinese herbal formula on POF through other ways.

Name	Formula	Hormone and related protein levels	Experimental model	Roles
Si Wu Tang (SWT) [67]	*Rehmannia glutinosa (Gaertn.)* DC. (Shu Di Huang) 12 g, *Angelica sinensis* (Oliv.) Diels (Dang Gui) 9 g, *Paeonia lactiflora Pall* (Bai Shao) 9 g, *Ligusticum striatum* DC. (Chuan Xiong) 6 g.	FSH ↓	Female C57BL/6 mice	SWT protected ovaries and improved ovarian angiogenesis by activating the Nrf2/HO-1 signaling pathway, the STAT3/HIF-1*α*/VEGF signaling pathway, and other proangiogenic factors.
		E2 ↑		
		SOD ↑		
		MDA ↓		
		Nrf2 ↑		
		HO-1 ↑		
		VEGF ↑ bFGF ↑		
		PDGFB ↑		
		Ang1 ↑ p-STAT3 ↑		
		STAT3 ↑		
		HIF-1*α* ↑		

Jiajian Guishen formula (JGF) [68]	*Cuscutae semen* (Tu Si Zi) 20 g, *Epimedii herba* (yin yang Huo) 15 g, *Rehmanniae preparata radix* (Shu Di Huang) 15 g, *Radix morindae officinalis* (Ba Ji Tian) 10 g, *Ginseng radix et rhizoma* (Ren Shen) 10 g, *Radix glycyrrhizae* (Gan Cao) 6 g	AMH ↑	SPF female ICR mice	JGF could improve the ovarian function of POI mice by suppressing SAHF.
		INHB ↑		
		HMGA1 ↓		
		HMGA2 ↓		
		H3K9me3 ↓		
		HP1 ↓		

## Data Availability

No data were used in this study.

## Abbreviations

POF: Premature ovarian failure
POI: Premature ovarian insufficiency
TCM: Traditional Chinese medicine
FSH: Follicle-stimulating hormone
E2: Estradiol
AMH: Müllerian hormone
LH: Luteinizing hormone
GC: Granulosa cell
JAK: Janus kinase
AQP8: Aquaporin 8
STAT: Signal transducer and activator of transcription
PF: Primal follicles
PI3K: Phosphoinositide 3-kinase
MTOR: Mechanistic target of rapamycin
OS: Oxidative stress
Cyt-c: Cytochrome C
ZGP: Zuogui pill
HYF: Huyang Yangkun formula
MBD: Modified Bazhen decoction
BJP: Bushen Jianpi prescription
BTR: Bushen Tianjing recipe
EXD: Er-Xian decoction
BHT: Bushen Huoxue formula
TSD: Taohong Siwu decoction
SWT: Si Wu Tang
JGF: Jiajian Guishen formula
TUNEL: Terminal deoxynucleotidyl transferase dUTP nick-end labeling
XIAP: X-Linked inhibitor of apoptosis protein
TNF-*α*: Tumor necrosis factor-alpha
VEGF: Vascular endothelial growth factor
NK cell: Natural killer cell
Th17 cell: IL-17-producing T helper cell
Rg1: Ginsenoside Rg1
BMP-15: Antibone morphogenetic protein 15
TGF-*α*: Transforming growth factor-*α*
SIRT1: Sirtuin-1
SAHF: Senescence-associated heterochromatic foci
HMGA: High mobility group A
INHB: Inhibin B
PDGFB: Platelet-derived growth factor subunit B
Ang: Angiotensin
HO-1: Heme oxygenase 1
BFGF: Basic fibroblast growth factor
H3K9me3: H3 lysine9 trimethylation
Nrf2: Nuclear factor E2 related factor 2
Keap1: Kelch-like ECH-associated protein 1
BIM: Bcl-2 interacting mediator of cell death
PGC1*α*: Peroxisome proliferator-activated receptor coactivator.
